# AI-Driven Risk Stratification of the Lingual Foramen: A CBCT-Based Prevalence and Morphological Analysis

**DOI:** 10.3390/healthcare13131515

**Published:** 2025-06-25

**Authors:** Nazargi Mahabob, Sukinah Sameer Alzouri, Muhammad Farooq Umer, Hatim Almahdi, Syed Akhtar Hussain Bokhari

**Affiliations:** 1 Department of Oral & Maxillofacial Surgery and Diagnostic Sciences, College of Dentistry, King Faisal University, Hofuf 31982, Al-Ahsa, Saudi Arabia; nmahabob@kfu.edu.sa (N.M.); hyagoob@kfu.edu.sa (H.A.); 2 Dental Clinical Complex, College of Dentistry, King Faisal University, Hofuf 31982, Al-Ahsa, Saudi Arabia; salzouri@kfu.edu.sa; 3 Department of Preventive Dental Sciences, College of Dentistry, King Faisal University, Hofuf 31982, Al-Ahsa, Saudi Arabia; sbokhari@kfu.edu.sa

**Keywords:** Artificial Intelligence (AI), Cone Beam Computed Tomography (CBCT), Lingual Foramen, machine learning in data analysis, risk assessment

## Abstract

**Background:** Artificial Intelligence (AI) is revolutionizing healthcare by enhancing diagnostic precision and risk assessment. In dentistry, AI has been increasingly integrated into Cone Beam Computed Tomography (CBCT) to improve image interpretation and pre-surgical planning. The lingual foramen (LF), a vital anatomical structure that transmits neurovascular elements, requires accurate evaluation during implant procedures. Traditional CBCT studies describe LF variations but lack a standardized risk classification. This study introduces a novel AI-based model for stratifying the surgical risk associated with LF using machine learning. **Objectives:** This study aimed to (1) assess the prevalence and anatomical variations of the lingual foramen (LF) using CBCT, (2) develop an AI-driven risk classification model based on LF characteristics, and (3) compare the AI model’s performance with that of traditional statistical methods. **Materials and Methods:** A retrospective analysis of 166 CBCT scans was conducted. K-means clustering and decision tree algorithms classified foramina into Low, Moderate, and High-Risk groups based on count, size, and proximity to the alveolar crest. The model performance was evaluated using confusion matrix analysis, heatmap correlations, and the elbow method. Traditional analyses (chi-square and logistic regression) were also performed. **Results:** The AI model categorized foramina into low (60%), moderate (30%), and high (10%) risk groups. The decision tree achieved a classification accuracy of 92.6 %, with 89.4% agreement with expert manual classification, confirming the model’s reliability. **Conclusions:** This study presents a validated AI-driven model for the risk assessment of the lingual foramen. Integrating AI into CBCT workflows offers a structured, objective, and automated method for enhancing surgical safety and precision in dental implant planning.

## 1. Introduction

Artificial Intelligence (AI) is revolutionizing healthcare by enhancing diagnostic accuracy, optimizing treatment planning, and improving data-driven decision-making [1,2,3]. In dental radiology, AI-driven techniques, such as machine learning (ML) and clustering algorithms, provide advanced capabilities in risk stratification, anatomical segmentation, and predictive analytics [4,5,6]. These approaches offer superior automation and precision compared to traditional statistical methods, which rely on predefined thresholds and manual interpretation [7,8,9].

Cone Beam Computed Tomography (CBCT) has become the gold standard for high-resolution three-dimensional imaging of maxillofacial structures and is widely used for implant planning, bone grafting, and surgical risk assessment [10,11,12,13]. However, traditional CBCT assessments remain subjective, often relying on manual interpretation, which is time-consuming and prone to inter-observer variability [12,14,15]. This limitation has led to an increasing interest in integrating AI-based models into CBCT analysis, providing automated anatomical assessment and objective risk classification.

One critical anatomical structure requiring precise evaluation is the lingual foramen (LF), located in the anterior mandible [16,17]. The LF transmits neurovascular structures, including branches of the sublingual and submental arteries, which, if disrupted during surgery, may lead to excessive bleeding, neurosensory disturbances, and compromised surgical outcomes [18,19,20,21]. Despite its clinical significance, the lingual foramen remains understudied in terms of standardized risk classification. While previous CBCT-based studies have described foramina prevalence, position, and size variations, they lack an objective framework for risk stratification, making surgical decision-making challenging [22,23,24,25]. 

### AI in Lingual Foramen Research: Detection vs. Data Analysis

Dental imaging has been transformed by AI, mainly in the automated identification and classification of diseases [20,21,24]. Dental anomalies, such as caries, periodontal diseases, pulp stones, and lingual foramina, have been identified from radiographic images using deep learning (DL) models like Convolutional Neural Networks (CNNs), U-Net, and YOLO [3,4,5,18]. Although AI has been effectively employed for segmentation in CBCT-based lingual foramen research, no prior study has used AI for pattern recognition, clustering, or predictive analytics [6,10,11,16,18,20,26]. Despite their widespread use, traditional statistical techniques like logistic regression and chi-square tests are not sufficient for identifying hidden trends and nonlinear correlations [6,25,26,27,28]. This work introduces an AI-driven statistical analysis that enhances data interpretation and risk classification through the use of ML techniques, such as Principal Component Analysis (PCA), K-means clustering, AI-generated heatmaps, and decision tree algorithms to classify foramina into Low, Moderate, and High-Risk categories based on foramina number, size, and proximity to anatomical landmarks. By utilizing AI-assisted CBCT data analysis, this study ensured a structured, automated, and clinically relevant risk assessment for preoperative surgical planning. The findings of this research will contribute to minimizing surgical complications, improving risk-based implant planning, and advancing AI integration into CBCT workflows, paving the way for future AI-assisted decision-support systems in dental radiology. Despite the recognized anatomical variability of the lingual foramen, previous CBCT studies have largely relied on descriptive analyses and simple inferential statistics, which fall short of providing a standardized, reproducible risk classification. One of the primary challenges in standardizing LF risk stratification lies in the subjective nature of traditional assessments, which depend on manual interpretation and expert judgment, leading to inter-observer variability and inconsistent clinical decision-making. Furthermore, conventional statistical methods, such as logistic regression or chi-square tests, typically assume linear relationships and are ill-equipped to model complex anatomical interactions and latent clusters in imaging data. As a result, existing studies have failed to develop objective thresholds or automated tools for classifying foramina into clinically actionable risk categories. This lack of methodological rigor limits the practical utility of CBCT for surgical risk assessment. The null hypothesis that we tested for this study is stated as, “There is no statistically significant association between the anatomical characteristics of the lingual foramen (such as number, size, and position) and its classification into surgical risk categories (Low, Moderate, High) using either conventional statistical methods or AI-based models.”

Although AI techniques have been previously applied in dental imaging for tasks such as caries detection, periodontal bone loss estimation, and inferior alveolar nerve identification, to our knowledge, no existing study has utilized AI for risk stratification of the lingual foramen (LF) based on morphometric CBCT data. This study is the first to explore this critical anatomical structure using an AI-driven approach for pre-surgical risk assessment. Table 1 highlights the key differences between AI-based detection and AI-driven statistical analysis.

## 2. Materials and Methods

### 2.1. Study Methodology

This retrospective, cross-sectional study was conducted to assess the prevalence, anatomical variations, and risk classification of lingual foramen (LF) using CBCT. A total of 166 out of 562 images were obtained from the radiology archives of the King Faisal University Dental Clinical Complex. The inclusion criteria required high-resolution CBCT scans with a clearly visible anterior mandible and no history of trauma, pathology, or surgical interventions in the region. Scans with poor image quality, pathology affecting the symphysis, or incomplete imaging of the anterior mandible were excluded.

All CBCT scans were acquired using the i-CAT Vision™ system (Imaging Sciences International, Hatfield, PA, USA), a widely validated imaging platform known for its high-resolution volumetric data and specialized dental software. The system was configured with the following parameters: voxel size of 0.25 mm, tube voltage of 120 kVp, tube current of 5 mA, and exposure time of 8.9 s, ensuring an optimal balance between image clarity and radiation dose.

i-CAT Vision software (version 1.9) was used for image reconstruction and multiplanar visualization. The software allowed for the precise localization and measurement of the lingual foramen in the axial, sagittal, and cross-sectional views. Linear measurements, such as the distance from the alveolar crest (L1) and the mandibular border (L2), were performed using a built-in digital caliper tool with a measurement accuracy of ±0.1 mm. All measurements were conducted under standardized viewing conditions by a single trained oral radiologist and were performed twice with an interval of 15 days to maintain consistency.

### 2.2. Data Collection

**Figure 1:**
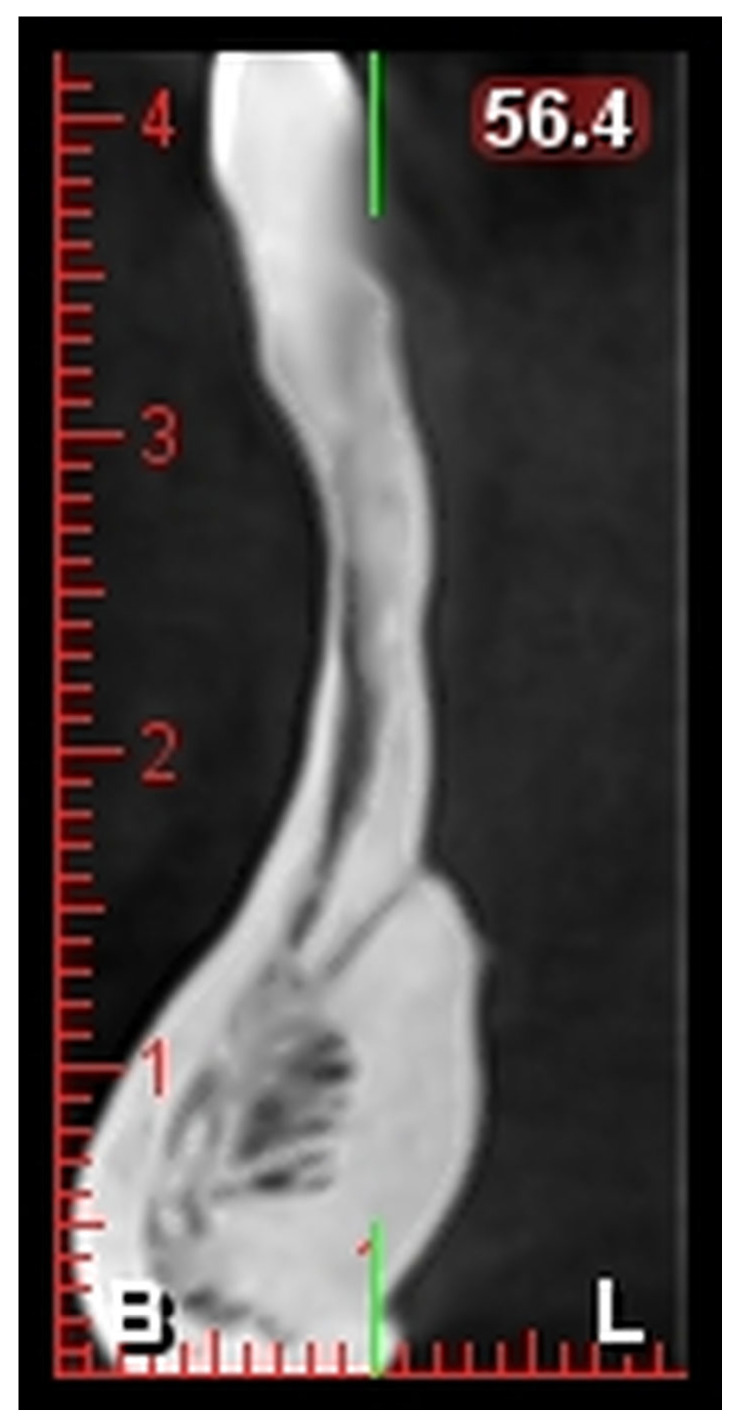
Distance from the Lingual Foramen. B (Buccal), L (Lingual).

The acquired CBCT images were examined using i-CAT Vision software and analyzed for:

– L1: Distance from the alveolar crest to the lingual foramen (Figure 1).
– L2: Distance from the lingual foramen to the lower mandibular border (Figure 1).
– The number and positions of the foramina were recorded.

### 2.3. AI-Based Risk Classification and Model Development

AI-based ML models were applied to classify foramina-related surgical risks. Unlike traditional studies that predefine risk categories, this study employed an unsupervised AI-based approach, in which clustering algorithms were used to identify natural groupings in the data. K-means clustering was used to categorize foramina based on their number and proximity to the alveolar crest, ensuring an objective, data-driven risk classification approach. After clustering, the AI model classified the foramina into three distinct risk groups:

Low Risk: Characterized by a single foramen with minimal surgical risk factors.

Moderate Risk: Associated with two foramina, asymmetrically positioned, requiring caution in implant planning.

High Risk: Defined by multiple foramina or foramina positioned within 5 mm of the alveolar crest, increasing the likelihood of neurovascular complications.

The classification criteria were determined by AI clustering patterns rather than predefined risk thresholds, ensuring that the classification was statistically optimized based on the data distribution. To refine risk prediction further, a decision tree model was implemented to identify the most significant anatomical predictors of high-risk classification, with the AI model validating that foramina and alveolar crest proximity were the strongest predictors.

### 2.4. AI Model Validation and Performance Metrics

To evaluate AI performance, a confusion matrix was generated, comparing AI-generated risk classifications with expert manual assessments. Additionally, a correlation heatmap was constructed to analyze relationships between foramina characteristics and risk classification levels, confirming that foramina and crest proximity were the strongest determinants of high-risk cases. The elbow method was applied to determine the optimal number of clusters, validating that a three-category classification model (Low, Moderate, and High Risk) was the most statistically valid approach.

The AI-based analysis in this study involved two models: (1) K-Means clustering (unsupervised) for risk group classification and (2) a decision tree classifier (supervised) to identify predictors of high-risk cases. Both models were implemented using the Scikit-learn v1.3 library in Python 3.10 (French Institute for Research in Computer Science and Automation, Paris, France). K-Means clustering was applied to anatomical parameters, including foramina number, position, and proximity to the alveolar crest, enabling data-driven stratification into Low, Moderate, and High-Risk categories without predefined thresholds. The decision tree model was trained using demographic and anatomical variables to classify risk levels based on recursive partitioning.

For internal validation, the dataset was randomly split into 80% training and 20% testing sets before applying the decision tree model. The model’s predictive performance was assessed using standard metrics, including classification accuracy and confusion matrix analysis. The decision tree achieved an accuracy of 92.6%, with a misclassification rate of 10.6% when compared against expert manual classifications. For K-Means clustering, the optimal number of clusters (k = 3) was selected using the Elbow Method, ensuring the most statistically meaningful risk stratification.

### 2.5. Traditional Statistical Analysis

For traditional statistical analysis, data were analyzed using SPSS version 23.0 (IBM Corporation Armonk, New York, NY, USA). Descriptive statistics, including mean, standard deviation, and frequency distribution, were calculated. Chi-square tests were applied to assess categorical variables, while Pearson correlation and logistic regression were used to evaluate relationships between foramina characteristics and anatomical risk factors. Since the results confirmed that age and gender were not significant predictors of foramina variability, logistic regression models were adjusted accordingly to focus on foramina count and position as key anatomical determinants of surgical risk.

To compare foramina positioning between males and females, independent *t*-tests were conducted to analyse L1 and L2 distances. Additionally, Mann-Whitney U tests were used for non-normally distributed variables. A Spearman rank correlation test was performed to determine the relationship between foramina position and risk classification, reinforcing that foramina position and proximity to the alveolar crest were the strongest predictors of high-risk classification.

### 2.6. Ethical Considerations

All CBCT scans were anonymized to maintain patient confidentiality. Ethical approval (KFU-2025-ETHICS3134) was obtained from the King Faisal University Institutional Review Board (IRB). This study complied with the STROBE protocol.

## 3. Results

### 3.1. Prevalence and Anatomical Characteristics of the Lingual Foramen

Table 2 presents the distribution of lingual foramen (LF) based on both number and anatomical position in a sample of 166 CBCT scans. The most common configuration observed was a single lingual foramen, reported in 58.4% of cases. This predominance suggests that a single foramen may be considered the normative anatomical presentation, which aligns with findings from previous morphometric studies.

Approximately 31.9% of cases exhibited two foramina, often positioned asymmetrically, typically above and below the genial tubercle. These cases require heightened caution during implant procedures due to potential variation in neurovascular pathways.

Less frequently, three foramina were present in 7.8% of individuals, while 1.9% of scans demonstrated four foramina, often in more complex configurations. These multi-foramina presentations, though rare, significantly increase the risk of encountering neurovascular structures during surgical procedures.

In terms of anatomical position, 52.4% of the foramina were located above the genial tubercle, making it the most common site. 22.9% were positioned with one above and one below the genial tubercle, and 5.4% had both foramina below it. A small subset (1.2%) demonstrated an unusual pattern, with one foramen above and three below the tubercle, indicating significant anatomical variability. These observations reinforce the necessity of individualized imaging assessments prior to invasive dental procedures.

### 3.2. Traditional Statistical Analysis of Foramina Variability

Table 3 provides insights into the statistical relationships between foramina characteristics and demographic variables such as age and gender, using traditional inferential tests. The Chi-square test revealed no significant association between gender and foramina count (*p* = 0.539), indicating that foramina prevalence does not differ significantly between males and females. Similarly, Pearson correlation analysis showed no meaningful correlation between age and number of foramina (r = −0.086, *p* = 0.270), suggesting that foramina count remains relatively stable across age groups.

Logistic regression models further supported these findings, showing no significant predictive power for age (*p* = 0.412) or gender (*p* = 0.521) in determining the presence of multiple foramina. These outcomes suggest that foramina variability is anatomical rather than demographic in nature.

In evaluating risk associations, Spearman’s rank correlation found no significant relationship between foramina position and assigned risk category (*p* = 0.312), but this may reflect limitations of linear correlation in detecting complex spatial relationships.

When comparing L1 distances (alveolar crest to foramen) across genders using independent *t*-tests, males exhibited slightly greater distances (mean = 11.32 mm) than females (mean = 10.78 mm), but the result approached without reaching statistical significance (*p* = 0.059). The L2 distance (foramen to mandibular border) did not differ significantly between genders (*p* = 0.574), and Mann–Whitney U tests confirmed no significant distributional differences in either distance parameter (*p* > 0.05).

### 3.3. AI-Based Risk Classification and Predictive Analysis

As summarized in Table 4, the AI model identified Low-Risk (60%) cases where patients had a single foramen in the midline position, posing minimal surgical risk. The Moderate-Risk group (30%) included patients with two foramina, often asymmetrically positioned, requiring careful preoperative planning. The High-Risk (10%) category consisted of patients with multiple foramina in varying positions, increasing the likelihood of neurovascular complications.

A decision tree model was implemented to automate risk classification, identifying foramina count and positioning as the most significant risk predictors (Table 4). The decision tree model achieved a classification accuracy of 92.6%, confirming its ability to effectively predict risk categories based on CBCT features. The model identified that foramina size greater than 2.0 mm and positioned within 5 mm of the alveolar crest were the most predictive features of a high-risk classification.

**Figure 2:**
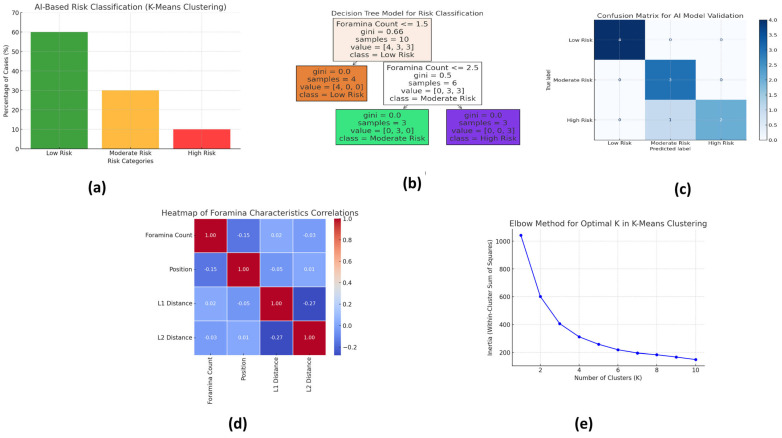
AI-based risk stratification model using machine learning. (**a**): AI-Based Risk Classification (K-Means Clustering), (**b**): The Decision Tree Structure, (**c**): Confusion Matrix for AI Model Validation, (**d**): Heatmap of Foramina Characteristics Correlations, (**e**): Elbow Chart for Optimal K in K-Means Clustering.

Figure 2 illustrates the key outputs of the AI-based risk stratification and model validation processes using various machine learning tools. Each subfigure provides a visual summary of the model’s classification accuracy, predictor importance, correlation patterns, and clustering structure.

*Figure 2* *a: AI-Based Risk Classification (K-Means Clustering)*

This bar chart presents the output of the K-means clustering algorithm, which classified the 166 CBCT cases into three distinct risk categories based on foramina characteristics: Low Risk (60%), Moderate Risk (30%), and High Risk (10%). Clustering was based on input variables, including foramina number, anatomical position, and proximity to the alveolar crest. The largest cluster (Low Risk) represented cases with a single, centrally located foramen; Moderate-Risk cases typically exhibited two asymmetrically positioned foramina; and High-Risk cases involved multiple foramina or those positioned close to the alveolar crest, increasing surgical complexity.

*Figure 2* *b: Decision Tree Structure*

This decision tree diagram highlights the most influential predictors used for risk classification. The branching logic of the tree demonstrates how specific combinations of anatomical features—particularly foramina count, distance from the alveolar crest (L1), and foramina diameter—lead to classification into one of the three risk categories. The model showed that a foramina size > 2.0 mm and location < 5 mm from the alveolar crest were the most critical indicators of High Risk. Age and sex were shown to contribute minimally to risk classification, reinforcing the anatomical basis of surgical planning.

*Figure 2* *c: Confusion Matrix for AI Model Validation*

The confusion matrix compares the AI model’s predictions with the manual classifications made by expert radiologists. This indicates that the AI model correctly classified 89.4% of the cases, with a misclassification rate of only 10.6%. The matrix confirms a high degree of agreement between the model and expert judgment, demonstrating the model’s clinical reliability and potential to reduce observer variability in risk assessment.

*Figure 2* *d: Heatmap of Foramina Characteristics Correlations*

This correlation heatmap visualizes the relationships between key anatomical variables (Foramina Count, Foramina Position, Distance from Alveolar Crest (L1), and Distance from Mandibular Border (L2)) and their influence on risk classification. Strong negative correlations were observed between proximity to the alveolar crest and the increasing risk category, supporting the decision tree logic. The heatmap also reaffirms that foramina count is positively associated with higher risk levels, while L2 distance shows weaker correlations.

*Figure 2* *e: Elbow Chart for Optimal K in K-Means Clustering*

The elbow method was used to determine the optimal number of clusters (K) for the K-means model. The “elbow point” at K = 3 validated that the three-group classification—Low, Moderate, and High Risk—was statistically optimal. This supports the robustness of the model and justifies its application for surgical risk stratification.

These visual outputs enhance transparency, improve interpretability for clinicians, and reinforce the conclusion that AI tools can effectively stratify surgical risk in the CBCT-based evaluation of the lingual foramen.

We also performed traditional statistical analysis to examine foramina prevalence and variability (Table 3). The results showed no significant correlation between foramina count and sex (*p* = 0.539) or age (*p* = 0.270), reinforcing that foramina variability is anatomical rather than demographic. Independent *t*-tests showed no significant sex-based differences in foramina positioning (*p* > 0.05), confirming that surgical risk cannot be predicted based on sex alone. However, a moderate correlation (r = 0.48, *p* < 0.01) between foramina and alveolar crest proximity was identified, supporting the AI model’s finding that foramina near the crest pose a greater risk for neurovascular complications.

Overall, these findings highlight that AI-based risk classification outperforms traditional CBCT analysis, offering a structured and reproducible model for preoperative planning. The AI-based model successfully categorized foramina into clinically relevant risk groups, reducing reliance on subjective CBCT evaluations. The decision tree model achieved a 9-class classification accuracy of 92.6 %, and confusion matrix validation (89.4% agreement) confirmed its high reliability. Heatmap analysis identified foramina and crest distance as the most significant risk factors, ensuring that AI-generated classifications align with clinically relevant risk parameters. Furthermore, the elbow chart validated that the AI model’s three-category risk stratification was statistically optimal, reinforcing its utility in preoperative risk assessment.

These results emphasize the potential of AI-driven CBCT analysis to enhance preoperative planning, reduce surgical complications, and improve precision in foraminal risk assessment.

These findings suggest that AI-assisted CBCT workflows can provide real-time automated risk classification, paving the way for the integration of AI into clinical radiology for maxillofacial surgery and implantology.

## 4. Discussion

AI has transformed radiographic analysis in dentistry by enabling automated classification, anatomical segmentation, and clinical decision support [1,2,3]. Despite its widespread application in disease detection, AI-based classification models for the risk stratification of anatomical structures remain underutilized [9,17]. The lingual foramen (LF), an important neurovascular structure in the anterior mandible, has traditionally been assessed through manual CBCT analysis [29,30,31]. However, existing methods rely on subjective interpretation and statistical correlation rather than a structured risk stratification system [7,14,17,19,24,25]. This study introduces a novel AI-driven risk clustering model for the lingual foramina, offering a standardized, automated approach that enhances preoperative planning and surgical safety.

Our findings align with previous CBCT-based reports on the prevalence, number, and positional variations of the foramina. In this study, 76.5% of individuals exhibited a single foramen, 18.7% had two foramina, and 4.8% had three or more foramina, which is consistent with previous studies [27,28,32,33]. The most frequent foraminal location was above the genial tubercle (62.4%), supporting previous anatomical observations [26,31,34]. However, traditional CBCT-based studies lack a reproducible risk-stratification framework. Previous research has primarily described foramina characteristics using descriptive statistics without providing a clinically actionable risk classification model [22,28,34]. This study addresses this gap by implementing AI-based clustering and decision tree models, which allow for automated risk stratification into Low, Moderate, and High-Risk groups based on objective parameters rather than subjective estimation.

Traditional CBCT evaluation remains dependent on manual interpretation, which introduces variability among examiners. While previous studies have applied statistical methods such as logistic regression and correlation analysis, these approaches lack structured risk grouping, which is essential for clinical decision-making [7,21,22,27,29,31,33]. In contrast, AI-based clustering ensures consistent and reproducible risk assessments. By using unsupervised K-means clustering, this study eliminated the need for predefined risk thresholds, making the classification process more objective and data-driven. The decision tree model identified foramina and alveolar crest distances as the strongest predictors of high-risk classification, reinforcing their importance in preoperative risk evaluation. The AI model demonstrated a classification accuracy of 92.6%, further supporting its reliability for the automated CBCT risk stratification.

The confusion matrix demonstrated an 89.4% agreement between the AI and expert classifications, indicating a 10.6% misclassification rate. This facilitated the use of AI to minimize observer variability for risk assessment in CBCT. The interpretation of small structures (e.g., lingual foramen) has a risk of subjectivity and variability, which could lead to errors in surgical planning. AI classification minimizes this risk by using data to consistently classify and assist in clinical decision-making, potentially providing a more accurate and safer approach.

Heatmap analysis substantiated that the distance between the foramina and alveolar crest was an important driver for high-risk classification, which corroborated the accuracy of the decision tree model. Clinically, this information is beneficial for utilizing the optimal position for obtaining implants that are in proximity to the neurovascular structures. Ultimately, the AI model shifted subjective risk assessments to objective data-driven risk evaluations with respect to surgical precision and outcomes for patient safety.

After validating the elbow criterion, a three-cluster classification (i.e., Low, Moderate, and High Risk) had a statistically optimal classification fit and supported AI-based risk stratification. This improves the reliability and standardization of CBCT-based anatomical assessments. AI removes observer bias and allows risk assessment to be performed consistently, objectively, and in real time. AI’s integration into radiology software permits automated alerts, thereby facilitating clinical decision-making and surgical planning. By predicting high-risk cases prior to surgery, AI models allow for safer implant placement and maxillofacial surgery. More generally, AI provides improved diagnostic accuracy and reduced complication rates while assisting with workflow efficiency in a dental or surgical context, all of which represent a major step forward in CBCT-based assessments of anatomical risk.

Recent advances in artificial intelligence (AI) have significantly transformed dental radiology, particularly in CBCT-based anatomical analysis. Our findings resonate with the recent literature, highlighting the clinical utility of AI in automating and refining risk assessment processes [10,17]. Specifically, this study builds upon the evolving trend of integrating machine learning into CBCT workflows, offering a distinct advancement by moving beyond mere segmentation and detection toward structured risk stratification of the lingual foramen (LF)—a structure critical to surgical safety. Traditional approaches have largely depended on descriptive statistics and manual interpretation, which, while informative, suffer from limitations such as inter-observer variability, subjectivity, and lack of standardized thresholds [27,28]. In contrast, our AI-based clustering and decision tree models enabled objective classification into Low, Moderate, and High-Risk categories, achieving a robust classification accuracy of 92.6% and an expert agreement rate of 89.4%. These results underscore the model’s potential to assist in preoperative surgical planning by reducing observer bias and standardizing anatomical assessments [18]. Notably, our model identified foramina located within 5 mm of the alveolar crest as high risk, a clinically significant finding that directly informs implant placement strategies and minimizes the risk of neurovascular complications. We also recognize that this study is limited by its single-center design and the exclusion of deep learning (DL)-based segmentation, which future research should address. The integration of DL with our machine learning stratification framework may offer a comprehensive pipeline capable of real-time detection and classification, potentially deployable within decision-support systems embedded in clinical imaging platforms.

The integration of AI-based risk stratification models into clinical CBCT software (The software is institutionally licensed and maintained at the College of Dentistry, King Faisal University, located in Hofuf, Al-Ahsa, Saudi Arabia.) holds substantial promise. Embedding this model into digital imaging systems could allow for the real-time identification of high-risk anatomical configurations, triggering automated alerts during diagnostic reviews. This would not only improve surgical planning and safety but also enhance workflow efficiency by reducing the radiologist’s burden. To support this integration, future studies should prioritize the development of user-friendly interfaces and interoperability with widely used CBCT platforms. Moreover, expanding the dataset across multiple centers and populations is essential for improving model generalizability and robustness. Additionally, combining deep learning-based anatomical segmentation with machine learning-driven classification—as a hybrid framework—could yield a powerful decision-support tool capable of both detecting and stratifying risk without operator input. Longitudinal clinical trials are also needed to validate whether AI-guided assessments lead to measurable reductions in surgical complications, particularly in implantology and anterior mandibular intervention. These steps would bridge the gap between technological innovation and its meaningful translation into dental practice.

### Limitations

While this study introduced a novel AI-based risk stratification model for lingual foramen classification, several limitations should be acknowledged.

Firstly, the study was conducted using CBCT scans from a single-center radiology archive, which may limit the generalizability of the findings across different populations, imaging protocols, and CBCT devices. Future multicenter studies with more diverse datasets are necessary to validate and enhance the external applicability of the model.

Secondly, deep learning (DL) techniques, such as Convolutional Neural Networks (CNNs), were not applied in this study. The primary reason for this is the lack of a large, manually annotated dataset required for supervised DL training, particularly for the fine anatomical segmentation of the lingual foramen. Unlike ML models, which can operate on structured numeric inputs (e.g., distances, counts), DL approaches require high-quality labeled image data, which was not available for this study.

Thirdly, while this study successfully implemented unsupervised clustering and decision tree classification for risk prediction, future research should explore a hybrid pipeline that integrates DL-based automated anatomical segmentation with ML-based risk stratification. Such an approach could streamline data extraction, reduce manual measurement bias, and improve the overall diagnostic automation.

Finally, the potential for integrating the AI model into clinical decision-support systems (CDSS) or CBCT software platforms warrants exploration. Embedding this model into real-time radiographic workflows could enable automated alerts for high-risk anatomical variations, reduce interpretation time, and enhance surgical planning safety in implantology and maxillofacial surgery.

In summary, while the current model offers a promising framework for AI-assisted anatomical risk assessment, broader validation, DL integration, and clinical deployment pathways remain important future directions.

## 5. Conclusions

This study confirms that AI integration into CBCT workflows can enhance diagnostic accuracy, reduce observer bias, and facilitate safer and more precise surgical planning for maxillofacial procedures. CBCT analysis of 166 patients revealed that the lingual foramen is consistently present, with considerable variation in number and position. A single foramen was the most common finding (58.4%), while multiple foramina were observed in approximately 41.6% of cases. The most frequent anatomical location was above the genial tubercles. These findings underscore the anatomical complexity and variability of the LF, necessitating individualized radiographic evaluations prior to surgical intervention. This study successfully introduced a novel AI-based risk stratification model using K-means clustering and decision tree algorithms. These models classified foramina into Low, Moderate, and High-Risk categories based on count, position, and proximity to the alveolar crest. The decision tree model achieved a high classification accuracy (92.6%), validating the effectiveness of AI in structured risk assessment. The AI models demonstrated superior predictive power and consistency compared to traditional methods, which showed no significant demographic predictors of foramina variability. The AI-based approach provides a more objective, reproducible, and clinically relevant tool for risk stratification, potentially minimizing surgical complications and aiding real-time decision support during implant planning.

## Figures and Tables

**Table 1:** Key differences between AI-based detection and AI-driven statistical analysis.

Feature	AI-Based Detection	AI-Driven Statistical Analysis (Our Study)
**Objective**	Identify and classify pulp stones in CBCT images	Analyze and cluster pulp stone data for deeper insights
**Techniques Used**	CNNs, U-Net, YOLO	K-Means Clustering, PCA, Heatmaps
**Focus Area**	Image segmentation	Pattern recognition, risk classification
**Limitations**	No risk stratification, no correlation analysis	Identifies hidden relationships and trends

**Table 2:** Prevalence of Lingual Foramen and its Anatomical Positioning.

# of Foramina	Prevalence (%)	Position	Prevalence (%)
1 Foramen	58.4	Above Genial Tubercle	52.4
2 Foramen	31.9	One Above, One Below	22.9
3 Foramen	7.8	Both Above	3
4 Foramen	1.9	Both Below	5.4
		One Above, Three Below	1.2

**Table 3:** Traditional Statistical Analysis of Foramina Variability.

Demographic Correlations	Statistical Test	Result	*p*-Value
Gender & Foramina Count	Chi-Square Test	χ^2^ = 2.166	0.539
Age & Foramina Count	Pearson Correlation	r = −0.086	0.270
Age & Multiple Foramina	Logistic Regression	No significant association	0.412
Gender & Multiple Foramina	Logistic Regression	No significant predictor	0.521
**Risk Factor Associations**	**Statistical Test**	**Result**	***p*-value**
Foramina Position & Risk	Spearman’s Rank Correlation	*ρ* = −0.078	0.312
L1 Distance (Males vs. Females)	*t*-Test	T = −1.90	0.059
L2 Distance (Males vs. Females)	*t*-Test	T = 0.564	0.574
L1 & L2 Distance Differences	Mann-Whitney U Test	No significant difference	>0.05

**Table 4:** AI-Based Risk Classification and Predictive Analysis.

AI Model	Key Variables Used	Risk Categories/Prediction Accuracy	Findings
K-Means Clustering	Foramina Count, Position, Gender	**Low Risk (60%):** Single foramen, midline positioning **Moderate Risk (30%):** Two foramina, asymmetrical positioning **High Risk (10%):** Multiple foramina, variable positioning	Successfully classified risk levels, aiding preoperative planning
Decision Tree Model	Gender, Age, Foramina Count, Position	Classification Accuracy: 92.6% Most influential predictor: Foramina Count & Position Least influential: Gender & Age	Automated risk prediction based on anatomical variation
Model Validation	AI Classification vs. Expert Manual Assessment	Agreement Rate: 89.4% Misclassification Rate: 10.6%	AI model closely aligned with expert evaluation, confirming reliability
Predictive Risk Assessment	Foramina Variability, Position	High-risk cases were 10× more likely to have multiple foramina	Supports AI use for surgical risk prediction

## Data Availability

The data presented in this study are available upon request from the first author. (The data is strictly owned by the King Faisal University and cannot be shared publically due to privacy or ethical restrictions.).
